# Therapy-resistant pretibial myxedema addressed with intralesional hyaluronidase

**DOI:** 10.1016/j.jdcr.2026.03.008

**Published:** 2026-03-13

**Authors:** Deborah Maria Gregersen, Susanne Darr-Foit, Jörg Tittelbach

**Affiliations:** Department of Dermatology (Klinik für Hautkrankheiten), University Hospital Jena (Universitätsklinikum Jena), Jena, Germany

**Keywords:** hyaluronidase, pretibial myxedema, triamcinolone

## Introduction

Myxedema is a rare and generally localized mucinosis characterized by the dermal accumulation of glycosaminoglycans (GAG), especially hyaluronic acid (HA). Pretibial myxedema (PM) has previously been shown to develop in approximately 0.5% to 4.3% of patients with Graves disease (GD). In most cases, euthyreosis does not lead to remission of PM nor improvement of associated endocrine orbitopathy (EO). Available treatment options for PM include topical corticosteroids, surgery, dermabrasion, physical therapies such as tissue and lymphatic drainage massage, and systemic therapies like octreotide and immunomodulators. However, despite the existence of various treatments options, the emergence of therapy resistance is a common challenge faced by patients and their treating clinicians.

Individual case reports have described successful treatment response in the application of intralesional triamcinolone, when it is combined with hyaluronidase. Notably, promising effects were also seen in the application of hyaluronidase on its own.^1^^,^^2^ Ophthalmologists have also used hyaluronidase injections to treat EO with encouraging outcomes.^3^ Nevertheless, no standardized treatment approach has been established, and disease control remains challenging to control.

## Case report

**Fig 1:**
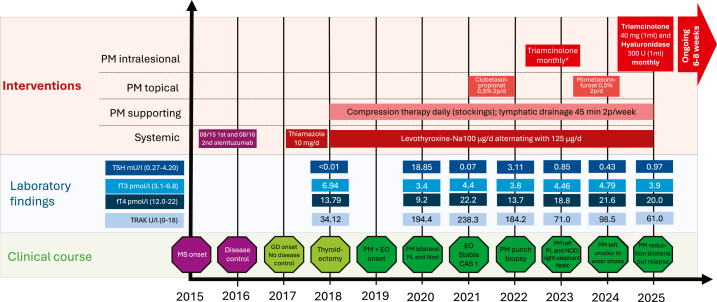
Case report timeline according to CARE guideline. *CAS*, Clinical activity score; *EO*, endocrine orbitopathy; *GD*, Graves disease; *MS*, multiple sclerosis; *NOD*, nodular; *PL*, plaque; *PM*, pretibial myxedema; *TRAK TSH*, receptor antibody; ∗no further details on the treatment regimen available.

We report on a 33-year-old woman treated with alemtuzumab for active progressive multiple sclerosis (MS) in 2016. After disease remission, she developed TRAK-positive GD in 2018 with a thyrotoxic crisis leading to thyroidectomy and iatrogenic hypothyroidism. Disease control was maintained by daily L-thyroxine substitution; however, TRAK positivity persisted (Fig 1).

Over the course of her disease, the patient gradually developed both periorbital and pretibial myxedema. Periorbital involvement was associated with the development of exophthalmos, whereas pretibial involvement was characterized by progressively increasing soft tissue proliferation. The EO remained clinically stable from 2 years after onset to the present (CAS 1/Hertel 16/16). Throughout follow up, TRAK levels remained markedly elevated. Thyroid function was generally maintained within the normal range under replacement therapy, although intermittent fluctuations were observed.

**Fig 2:**
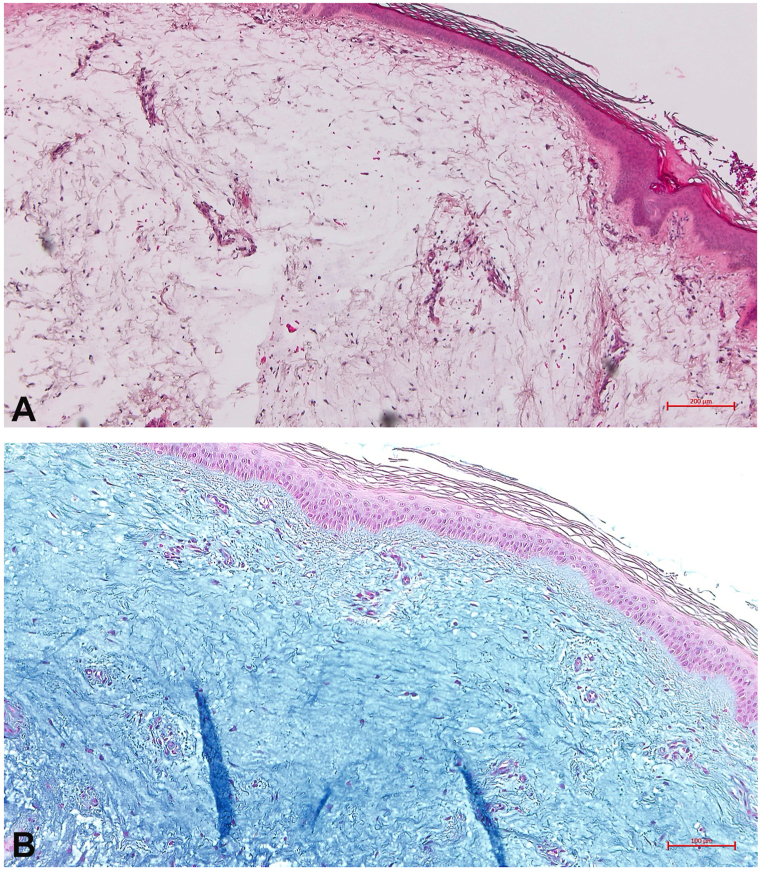
Detection of dermal interstitial mucin deposits, which appear as optically empty spaces in **(A)** H&E staining (×50); visualized in **(B)** basic Alcian blue staining (×100).

PM initially manifested as well-demarcated plaques and nodular lesions. With disease progression, the right pretibial plaque evolved into an elephantiasic form with marked tissue enlargement. To exclude malignant or lymphatic conditions, a skin biopsy was performed in 2022. Histopathologic examination was able to rule out B cell lymphoma and papillomatosis cutis lymphostatica, and revealed perivascular lymphocytic infiltrates and numerous fibroblasts within abundant Alcian blue-positive mucin deposits. These findings were consistent with GD-associated PM (Fig 2).

**Fig 3:**
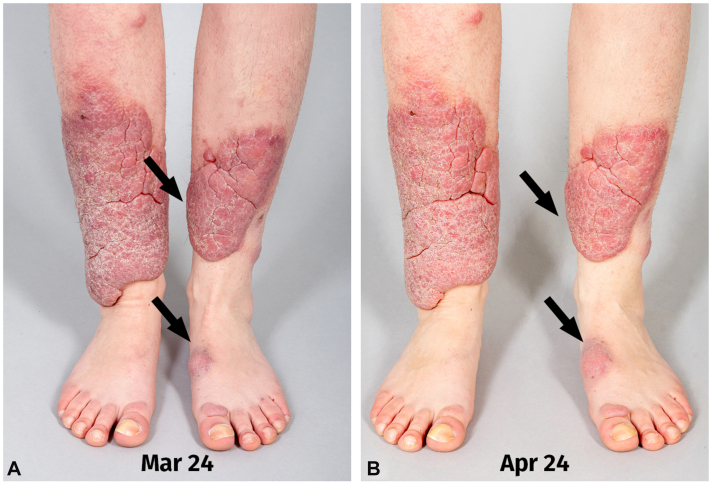
Rapid progression of lesions from March 2024 **(A)** to April 2024 **(B)**, with prominent growth on the back of the left foot, making it increasingly difficult to wear shoes. The *arrows* indicate the locations of the most prominent growth, which was also where the functional impairment was greatest.

Two years of compression therapy (class II stockings daily) and manual lymphatic drainage (twice a week for 45 minutes) combined with intralesional triamcinolone acetonide monotherapy (40 mg every 4 weeks) resulted in no clinical improvement. The patient presented in spring 2024 with marked progression of bilateral pretibial lesions and a nodular lesion on the left foot that impaired the ability to wear shoes (Fig 3).

After discussing treatment options, we initiated off-label treatment combination therapy with intralesional triamcinolone acetonide and hyaluronidase administered monthly. We selected HYLASE “Dessau” 300 IU because it was the only hyaluronidase formulation available at the time treatment was initiated. HYLASE “Dessau” contains small amounts of bovine-derived gelatin hydrolysate. The patient had no history of allergies, therefore we did not suspect hypersensitivity and refrained from performing prior skin testing, despite this being recommended in the product information sheet.

Both medications, HYLASE “Dessau” 300 IU and triamcinolone acetonide 40 mg/mL, were prepared in accordance with the product information by reconstituting each in 1 ml of 0.9% NaCl solution. The 2 medications were then drawn into a 1 ml tuberculin Luer lock syringe in a 1:1 ratio.

After 15 minutes of antisepsis, 0.1-ml aliquots were injected at 1-cm intervals. We targeted the center of the myxedema plaque using a combination of linear and fan-shaped injection techniques, adjusted to the anatomy and plaque characteristics. Injections always began in the thickest and most distal areas. A long needle (40 mm, 18-gauge) and a short needle (25 mm, 27-gauge) were used depending on plaque size and firmness. Injection points varied between sessions due to anatomic differences.

Each pretibial plaque was treated with 1 ml of solution containing 150 IU of HYLASE “Dessau” and 20 mg of triamcinolone acetonide. Any post-injection bleeding was covered with a bandage, and the patient was discharged 30 minutes after the procedure. No anaphylactic reactions occurred during the treatment period. Treatment led to arrest of disease progression within 1 month and gradual lesion regression. Regular shoes were able to be worn again after 2 months treatment.

Side effects included burning and pressure sensations at the injection sites. We observed the intended atrophy of the PM plaques and nodules. Other glucocorticosteroid-associated adverse effects such as pigmentary changes were not observed. After 3 months, the patient reported irregular menses during 2 cycles. Although we cannot rule out a glucocorticosteroid effect, the patient had previously tolerated 1 year of monthly triamcinolone injections without menstrual irregularities. We therefore continued treatment, as menses subsequently normalized, suggesting no causal relation to triamcinolone.

**Fig 4:**
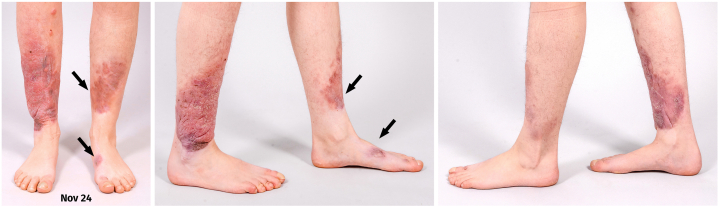
November 2024: Significant improvement achieved with our treatment plan; the left leg showed a better response compared to the right leg. The *arrows* indicate the locations of the most prominent growth, which was also where the functional impairment was greatest.

**Fig 5:**
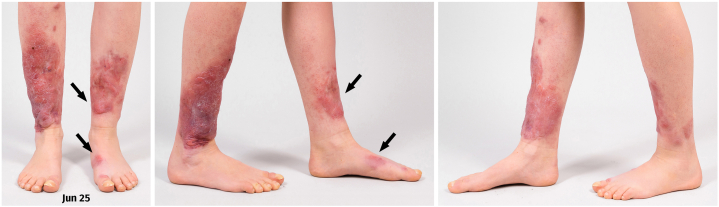
March 2025 end of treatment: Further involution is obtained, nearly complete involution of the plaque on the left foot. The *arrows* indicate the locations of the most prominent growth, which was also where the functional impairment was greatest.

After 12 months, therapy was paused for follow-up. Although complete remission was not achieved, significant lesion involution was observed, with approximately >90% reduction of PM in the left plaque and >50% reduction in the right plaque (Figs 4 and 5 and Table I). Compression therapy and lymphatic drainage remain ongoing.

**Table I:** Approx. PM sizes in cm per timepoint and localization

	Left foot nodule [cm]	Left PM plaque [cm]	Right PM plaque [cm]
APR 24	5 × 3 × 1.5	10 × 12 × 1.5	15 × 15 × 2.5
MAR 25	2.5 × 2 × 0.3	9 × 6 × 0	15 × 13 × 1

**Fig 6:**
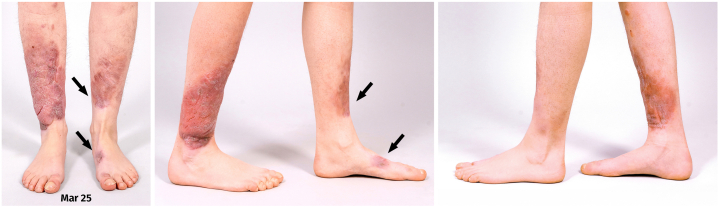
June 2025: Relapse on both sides with recurring nodules on the treated plaques. The *arrows* indicate the locations of the most prominent growth, which was also where the functional impairment was greatest.

Three months post-treatment, a nodular relapse occurred bilaterally (Fig 6 and Table I). We resumed injections at extended intervals (every 6 to 8 weeks). Early follow-up showed cessation of new lesion formation and again encouraging regression.

## Discussion

This case illustrates substantial improvement of therapy-resistant PM following combined intralesional triamcinolone and hyaluronidase (as described by Kudlak et al, 2020)^2^ after failure of triamcinolone monotherapy and limited efficacy of hyaluronidase monotherapy (as described by Hoesly et al, 2018).^1^

Hyaluronidase increases extracellular matrix permeability and reduces connective tissue viscosity by hydrolyzing mucopolysaccharides such as hyaluronic acid and chondroitin sulfates. This so-called spreading factor accelerates fluid absorption post-injection. The anti-inflammatory and antifibrotic agent triamcinolone is likely to achieve improved tissue distribution and exert inhibitory effects on activated fibroblasts. Therefore, both agents act on the inflammatory milieu in GD, where autoantibodies stimulate fibroblast GAG production and cytokine release thereby maintaining lymphocytic infiltration. Plaque reduction may result not only from GAG breakdown but also from the antiedematous effects of the corticosteroid. The nearly immediate arrest of disease activity observed in our patient supports this hypothesis. Anti-inflammatory effects may also target IGF-1R-activated keratinocytes and fibroblasts. Recently, upregulation of IGF-1R in patients with GD has been demonstrated not only in fibroblasts but also in keratinocytes of patients with PM. The FDA-approved IGF-1R antagonist teprotumumab for GD-associated EO has shown beneficial effects on PM as well. Therefore, IGF-1R antagonism has been suggested as a novel therapeutic target for PM,^4^ although such therapies remain experimental.

Hyaluronidase is available as a recombinant bacterial enzyme or as an enzyme purified from ovine or bovine sources; the latter requires allergy screening due to the risk of anaphylaxis. In our patient, a clear medical history did not indicate any suspicion of allergy; therefore, we opted not to perform prior skin testing as previously described.^1^ In the event of potential anaphylaxis, trained healthcare professionals and emergency medications were always available.

We believe that additional physical therapy and compression therapy support tissue reduction especially in combination with hyaluronidase, given its ability to reduce tissue viscosity and enhance permeability.

The PM on the left leg showed faster tissue reduction than on the right leg, where the PM plaques were more prominent. In 1 case report, no response was observed in the right extremity.^1^ Possible explanations in our case include differences in plaque size, chronicity and degree of induration, despite equal treatment with 1 ml of hyaluronidase and triamcinolone in both pretibial regions. Whether a general pattern regarding treatment response or failure in GD-associated pretibial myxedema exists cannot be determined based on only 2 reported cases.

The criteria for starting treatment—both initially and during relapse—were pain, disease activity, and loss of function. After the relapse, we resumed treatment using the same doses that had previously been effective. Our main treatment approach is to use the lowest effective dose that arrests disease activity and reduces pretibial myxedema. In this case, with controlled but chronically active GD we aimed to extend the interval between treatments to reduce the cumulative dose. Nevertheless, the disease course is unpredictable, and chronic progression is expected.

We attributed the patient’s relapse primarily to the degradation of triamcinolone, which reduced its intralesional anti-inflammatory and antifibrotic effects. TRAK antibodies have remained elevated at fluctuating levels. This might account for ongoing disease activity and contribute to relapse, although the TRAK level was not particularly high at the time of relapse. To date, the EO remained stable since 2020, suggesting that unknown factors may account for disease activity confined to the pretibial region.

Given the rarity and disfiguring nature of PM, no standard therapy exists. Compared to systemic or surgical options, topical or intralesional treatments offer better tolerability. Our findings support earlier suggestions that combined triamcinolone and hyaluronidase represent a safe and potentially effective therapeutic option.

## Conflicts of interest

None disclosed.
